# Dr. George H. Cushing

**Published:** 1900-07

**Authors:** 


### DR. GEORGE H. CUSHING.

THE death of this prominent member of the dental profession, while not entirely unexpected, has created a deep feeling throughout dental circles.

Dr. Cushing was born in 1829 in Providence, R. I. His father was for sixty years the cashier of a bank in the city.

After finishing his professional studies, Dr. Cushing went to California soon after the discovery of gold, and spent a year or two prospecting and mining. Returning thence, he came to Chicago about 1856, beginning the practice of his profession in partnership with the Quinlan Brothers, then the leading dentists in the city, succeeding to their practice on their retirement soon after.

Dr. Cushing continued the practice of his profession in Chicago until 1898. His office was destroyed in the great fire in October, 1871, but his house in Lake View was outside the burnt district. He leaves a widow, two sons, and two daughters.

Dr. Cushing's health was so impaired some two years previous that it necessitated a change of climate, and he removed from Chicago to California. This appeared to benefit him so much that he was able to attend the meeting of the National Dental Association held at Niagara Falls in 1899, and he was then apparently restored to his usual health and spirits. His friends were, therefore, not prepared for the telegraphic announcement of his death.

Dr. Cushing's work has been almost entirely confined to Chicago as a practitioner and to his labors as secretary of the American Dental Association. He was an active member of this body from its earlier organization, and became its President in 1871, being elected to that position at White Sulphur Springs, and acting in that capacity at Niagara Falls in 1872. He was subsequently elected secretary, and filled this position to the great satisfaction of the Association until the final meeting of that body in 1897, and was then made secretary of the new organization, the National Dental Association, and continued in this service up to the time of his last illness.

It is probably true that but few men were better known in the dentistry of this country. While his work was mainly confined to the active duties of the associations with which he was connected, these were so important and so satisfactorily performed and so long continued that he became one of the chief standard-bearers of his profession, always to be depended upon to carry out his arduous duties with fidelity to the trust imposed upon him.

His personality attracted a large circle of friends, and probably few men would have been able to cover his manifold duties with less criticism. His friends were not merely such by name, but were closely attached to him by ties of a character few may be able to claim; and when sickness made him unfit for further practice these manifested their interest in ways that must have been a consolation in the closing period of his active career.

His life covered the most interesting period in the dental profession. He came into it at the transition decade, and, living to the last year of the century, he departed with the assurance that it had reached a standard beyond his or others' fondest anticipations.

While we mourn his loss as a friend and earnest associate, we feel that his example will live as an incentive to a younger generation. The curtain is being rapidly run down upon the life scenes of the older workers; one by one they are dropping from the active duties of life, but the lessons they taught must be our sure inheritance, to remain with us and be an inspiration to work worthily in their paths and extend these through the tangled undergrowth of, as yet, many unexplored regions to the clearer and unobstructed atmosphere of a higher professional life.

